# Does length of time since diagnosis in Parkinson’s disease influence heart rate variability? A cross-sectional study

**DOI:** 10.1590/0004-282X-ANP-2021-0153

**Published:** 2022-05-20

**Authors:** Mileide Cristina Stoco-Oliveira, Heloisa Balotari Valente, Laís Manata Vanzella, Larissa Borba André, Mariana Viana Rodrigues, Franciele Marques Vanderlei, Augusto Cesinando de Carvalho, Luiz Carlos Marques Vanderlei

**Affiliations:** 1 Universidade Estadual Paulista “Júlio de Mesquita Filho”, Faculdade de Ciências e Tecnologia, Departamento de Fisioterapia, Presidente Prudente SP, Brazil.; 2 University Health Network, Toronto Rehabilitation Institute, East York ON, Canada.

**Keywords:** Parkinson Disease, Autonomic Nervous System Diseases, Neurodegenerative Diseases, Doença de Parkinson, Doenças do Sistema Nervoso Autônomo, Doenças Neurodegenerativas

## Abstract

**Background:**

Intrinsic changes in Parkinson's disease (PD) affect the autonomic nervous system, and the disease course can aggravate the initial condition. Although the impact of time since disease onset on autonomic modulation has already been studied in other populations, this has not yet been investigated in PD.

**Objective:**

To investigate the impact of the length of time since diagnosis on the cardiac autonomic modulation of individuals with PD and compare with healthy individuals.

**Methods:**

Fifty participants were divided into three groups: a control group (CG; n = 24) and two groups with PD, divided according to the median length of time since diagnosis (median = 5.5 years): below the median (PG1; n = 13) and above the median (PG2; n = 13). To evaluate cardiac autonomic modulation, heart rate was obtained beat-to-beat in the supine position over a 30-min period, and heart rate variability (HRV) indices were calculated using linear methods in the time and frequency domains.

**Results:**

There were no significant differences in HRV indices between the PG groups, or between the three groups regarding Mean RR, LFun, HFun and LF/HF ratio. Significant reductions in the RMSSD, SDNN, pNN50, LFms^2^ and HFms^2^ indices were observed in PG1 and PG2, compared with CG.

**Conclusions:**

The cardiac autonomic modulation of individuals with PD was not influenced by the time since diagnosis. However, reduced parasympathetic and global modulation were observed in these individuals, compared with controls. These results emphasize the importance of aerobic exercise for improving autonomic modulation among individuals with PD.

## RESUMO

**Antecedentes:**

As alterações intrínsecas da doença de Parkinson (DP) afetam o sistema nervoso autônomo, e a evolução da doença pode agravar o quadro inicial. Em outras populações, o impacto do tempo desde o início da doença na modulação autonômica já foi estudado, mas na DP isso ainda não foi investigado.

**Objetivo:**

Investigar o impacto do tempo de diagnóstico na modulação autonômica cardíaca de indivíduos com DP e comparar os valores aos de indivíduos saudáveis.

**Métodos:**

Cinquenta participantes foram divididos em três grupos: grupo controle (GC; n=24) e dois grupos com DP, divididos de acordo com a mediana do tempo de diagnóstico (5,5 anos): abaixo (GP1; n=13) e acima da mediana (GP2; n=13). Para a avaliação da modulação autonômica cardíaca, a frequência cardíaca foi captada batimento a batimento em posição supina durante 30 minutos, e os índices de variabilidade da frequência cardíaca (VFC) foram calculados utilizando métodos lineares nos domínios do tempo e frequência.

**Resultados:**

Não houve diferenças significativas para os índices de VFC entre os grupos GP, ou entre os três grupos para Mean RR, LFun, HFun e relação LF/HF. Foram observadas reduções significativas em RMSSD, SDNN, pNN50, LFms^2^ e HFms^2^, para GP1 e GP2 em comparação ao GC.

**Conclusões:**

A modulação autonômica cardíaca de indivíduos com DP não foi influenciada pelo tempo de diagnóstico, contudo, foi observada redução da modulação parassimpática e global nesses indivíduos em relação aos controles. Esses resultados reforçam a importância do exercício aeróbio para a melhora da modulação autonômica de indivíduos com DP.

## INTRODUCTION

Parkinson's disease (PD), characterized by death of dopaminergic neurons located in the substantia nigra pars compacta^1^, is considered to be the second most common neurodegenerative disease worldwide^2^. Its incidence in individuals aged over 50 years is increasing, such that it is expected to reach between 8.7 and 9.3 million people by 2030^3^. 

 The cardinal motor symptoms of PD are postural instability, bradykinesia, rigidity and resting tremor^4^. During the course of the disease, abnormalities related to the autonomic nervous system (ANS)^5^ may also be observed, which further worsen the overall clinical condition and lead to significant worsening of quality of life^6^. ANS alterations can be evaluated through heart rate variability (HRV)^7^, a non-invasive method in which sinus beat intervals RR intervals) are analyzed. These intervals are associated with the influences of the ANS on the sinus node^7^. Studies using this analysis among individuals with PD have demonstrated that HRV is lower in this population^8^ ^,^ ^9^ ^,^ ^10^. This is an autonomic dysfunction that could be a consequence of autonomic regulatory degeneration in the brain and peripheral autonomic ganglia^9^.

Several conditions may influence HRV, such as metabolic alterations^11^, body composition^12^, age^13^, cardiovascular risk factors^12^ and pathological conditions^11^ ^,^ ^12^ ^,^ ^14^. Specifically in PD, the stage of the disease^15^, body mass index^16^ and use of levodopa medication^17^ may also influence HRV. However, through searching the literature, we were unable to find any studies that evaluated possible influences from the length of time since the diagnosis of PD was made, on autonomic dysfunctions. 

In the literature, autonomic impairments in the PD population have been described. However, this raises a number of questions: Does the length of time since diagnosis influence the cardiac autonomic modulation of this population? Do individuals with longer times since diagnosis present worse cardiac autonomic modulation than individuals with shorter times? Does the cardiac autonomic modulation of individuals with PD with longer or shorter times since diagnosis differ from that of individuals without the disease? To fill these gaps in knowledge, the aim of the current study was to evaluate the impact of the length of time since diagnosis on the cardiac autonomic modulation of individuals with PD and compare these values with those of individuals without the disease.

The hypothesis of this study was that the length of time since the diagnosis of PD was made influences cardiac autonomic modulation, such that individuals with longer times since diagnosis would present worse cardiac autonomic modulation, and that these differences would be greater than those among healthy individuals. Understanding these matters is important for clinicians and researchers, given that these results could aid in elaboration of treatments aimed at promoting increased cardiac autonomic modulation and, thus, could reduce the risks induced in individuals with PD through autonomic alterations. 

## METHODS

### Study design and ethical matters

This cross-sectional observational study was reported in accordance with the STROBE guidelines. The study was conducted in Presidente Prudente, São Paulo, Brazil, between August 2017 and April 2018. All procedures used were approved by the University’s Human Ethics Committee. The participants were informed about the procedures and objectives of the study and, after agreeing to participate, provided written informed consent.

### Population

The participants with PD were recruited from the neurology sector of the Center for Physical Therapy and Rehabilitation Studies and Treatment of São Paulo State University (UNESP) Faculty of Sciences and Technology, Presidente Prudente, Brazil, and the matching controls were recruited from health care centers and clinics in the same city. The participants with PD were required to have a medical diagnosis of PD, based on the presence of the clinical criteria^18^, independent of the length of time since diagnosis, and to be classified in stages 1 to 3 of the *Hoehn and Yahr* (HY) scale^19^. The PD participants were divided into two groups according to the median length of time since diagnosis (median = 5.5 years): a group below the median (PG1; n = 13 participants) and a group above the median (PG2; n = 13 participants). Participants without the disease were considered for inclusion in the control group (CG; n = 24 participants) and were paired with individuals in the PG groups according to age. 

The participants were required to present an absence of cognitive deficits, as evaluated though the Mini-Mental State Examination (MMSE)^20^, in order to ensure understanding of the procedures performed during data collection. Current smokers, current heavy drinkers, individuals with active infections, cognitive deficits or cardiovascular and respiratory diseases and individuals who did not sign the informed consent statement were not included in the study. Participants with more than 5% error in the RR interval series recordings were excluded. 

The sample size was based on the Root mean square of differences between adjacent normal RR intervals in a time interval, expressed in ms (RMSSD index). The significant difference assumed was 9ms and standard deviation 3ms with the number of participants analyzed, and a significance level of 5% (two-tailed), confirming a power > 80% to detect differences between the variables.

### Study design

The study was divided into two steps, with intervals ranging from 24 hours to one week between them. Data collection was performed during the “on” period of levodopa medication of the participants with PD^21^. In the first step, personal data (to investigate the inclusion and exclusion criteria and identify age, sex, use of medication and length of time since diagnosis), physical parameters (body composition) and clinical parameters (cardiovascular parameters of heart rate and blood pressure; PD stage; and cognition evaluation) were obtained. In the second stage, cardiac autonomic modulation was evaluated.

The data collection was performed in a room with a temperature of between 21 and 23 °C and humidity of 40 to 60%, at times between 8 am and 12 pm to minimize the influence of circadian rhythm^22^. The assessment was performed individually, and the participants were instructed not to consume alcohol and/or stimulant substances, such as coffee, tea, chocolate and soda, or perform physical exercise, for 12 hours prior to the assessments.

### Experimental procedures

*First step*

After personal data had been collected and the cognitive assessment had been performed using the MMSE^20^, body composition (height, weight and body mass index, BMI), cardiovascular parameters and PD stage were evaluated.

Body composition

To assess body composition, the participants were asked to wear appropriate clothes and no shoes. Height was measured using a stadiometer (Sanny; São Paulo, Brazil) and body weight was measured using a digital scale (Welmy R/I 200; Santa Bárbara D’Oeste/SP, Brazil). BMI was calculated using the following formula: weight/height^2^ (kg/m^2^)^23^.

Body fat and lean mass were obtained through a Maltron bioimpedance device (Maltron BF 906 Body fat analyzer; Maltron, UK)^24^.

Cardiovascular parameters

Systolic (SBP) and diastolic (DBP) blood pressures were indirectly measured using a stethoscope (Littman; Saint Paul, Minnesota, USA) and an aneroid sphygmomanometer (WelchAllyn - Tycos; New York, USA) on the left arm^25^. The resting heart rate was measured using the same heart rate monitor used for HRV assessment (Polar RS800CX, Polar Electro; Kempele, Finland).

Parkinson’s disease stage

To determine the PD stage, the HY scale was used^19^. The classification of individuals with PD was made by a physiotherapist with specialization in neurology and in treatment of these individuals. 

*Second step*

Cardiac autonomic modulation

To analyze cardiac autonomic modulation, heart rate was recorded beat-to-beat using a Polar RS800CX heart rate monitor (Polar, Finland). For the recording, the participants remained in a supine position for 30 minutes, while breathing spontaneously but avoiding conversation, during the procedure.

### Outcomes

*Cardiac autonomic modulation*

The series of RR intervals was subjected to digital filtering using the Polar Precision Performance SW software (version 4.01.029), followed by manual filtering performed through the Excel software, to eliminate ectopic premature and artifact beats. Only series with more than 95% sinus beats were included in the study^26^. Cardiac autonomic modulation was analyzed using 1000 consecutive RR intervals, obtained from the most stable part of the series^26^. The Kubios HRV software, version 3.1, was used to calculate the HRV indices^27^. 

To analyze HRV in the time domain, the indices Mean RR, rMSSD, SDNN and pNN50 were used. Mean RR represents the mean value of the RR intervals. rMSSD is the root mean square of differences between adjacent normal RR intervals in a time interval, expressed in ms^7^. SDNN is the standard deviation of all normal RR intervals, expressed in ms. pNN50 is the percentage of adjacent RR intervals with a difference in duration > 50 ms^7^.

 For analysis on the frequency domain, the spectral components of low frequency (LF; 0.04 to 0.15 Hertz) and high frequency (HF; 0.15 to 0.4 Hertz), expressed in milliseconds squared (ms^2^) and normalized units, and the LF/HF ratio, were calculated using a fast Fourier transform algorithm^7^.

### Data analysis

The normality of the data was tested using the Shapiro-Wilk test. A descriptive statistical method was used for data presentation, and the results were presented as means and standard deviations (for parametric data), medians and interquartile ranges (for nonparametric data) and confidence intervals, absolute frequencies and relative frequencies (for qualitative data). Sample characterization data and HRV indices were compared between the groups using covariance analysis (ANCOVA), adjusted for sex and BMI. Possible differences were assessed using the Bonferroni post-test. Data on medicines in use were compared using the chi-square test (Yates's correction was applied in 2 x 2 contingency tables). 

The effect size of the differences between the groups was measured using partial eta squared. The effect size was defined as low (≤ 0.01), moderate (0.06 to 0.14) or high (≥ 0.14)^28^. The significance level was set at 5%. The analyses were performed using SPSS version 15.0 (SPSS Inc.; Chicago, IL, USA).

## RESULTS

**Figure 1.**
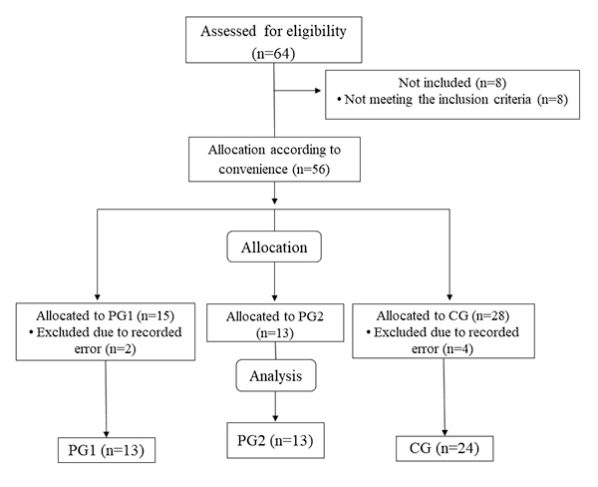
Flow diagram.

The distribution and sample losses during the steps of the study are demonstrated in Figure 1. 

Table 1 presents the characteristics of the three groups studied and Table 2 demonstrates the medicines used by the participants. In Table 1, significant differences were observed for DBP, length of time since diagnosis and MMSE (p < 0.05). The groups were classified as overweight^23^, pre-hypertension^25^ and absence of cognitive deficits^20^. In addition, most of the participants with PD were classified as having stage two of the disease^19^. In Table 2, significant differences were observed with regard to dopamine receptor blockers, levodopa and beta-blockers. 

**Table 1.** Characterization of the control group (CG) and Parkinson’s groups (PG1 and PG2) evaluated in this study.

	CG (n = 24)	PG1 (n = 13)	PG2 (n = 13)	P value
Age (years)	70.25 ± 8.03 66.86-73.64	70.23 ± 8.30 65.22-75.25	75.23 ± 6.09 71.64-79.01	0.13
SBP (mmHg)	130.42 ± 13.34 124.78-136.05	123.85 ± 10.44 117.54-130.15	128.46 ± 13.45 120.34-136.59	0.33
DBP (mmHg)	85.00 [10.00] 83.00-90.33	79.23 ± 9.54^a^ 73.47-85.00	80.00 ± 11.55 73.02-87.08	0.045
HR (bpm)	63.50 [11.25] 57.29-68.21	64.15 ± 6.94 59.96-68.35	65.69 ± 11.10 59.08-72.40	0.75
BMI (kg/m^2^)	29.43 ± 4.05 27.72-31.14	27.76 ± 3.11 25.88-29.63	26.39 ± 4.79 23.49-29.28	0.09
Body fat (%)	32.09 ± 9.58 28.06-36.12	31.98 ± 8.35 26.93-37.03	34.11 ± 9.10 28.61-39.61	0.78
Lean mass (%)	67.84 ± 9.48 63.83-71.84	68.02 ± 8.35 62.97-73.06	65.89 ± 9.10 60.39-71.39	0.79
Length of time since diagnosis (years)	—	2.62 ± 1.61 1.64-3.59	8.00 [7.50]^b^ 7.75-13.33	<0.0001
HY scale	—	2.00 [1.00] 1.74-2.57	3.00 [1.00] 2.22-2.85	0.12
MMSE	27.50 [3.00] 25.56-28.19	28.00 [4.50] 23.39-28.30	23.31 ± 4.66^a^ 20.50-26.12	0.03

Mean ± standard deviation; lower boundary - upper boundary of 95% confidence interval; median [interquartile range]; ^a^value with difference in relation to control group; ^b^value with difference in relation to PG1; CG: control group; PG1: Parkinson group below the median; PG2: Parkinson group above the median; SBP: systolic blood pressure; DBP: diastolic blood pressure; HR: heart rate; BMI: body mass index; mmHg: millimeters of mercury; bpm: beats per minute; kg: kilogram; m: meters; m^2:^ square meters; MMSE: mini-mental status examination; HY: Hoehn and Yahr; %: percentage.

**Table 2.** Medication in use by the volunteers in the control group (CG) and Parkinson’s groups (PG1 and PG2) evaluated in this study.

	CG (n = 24)	PG1 (n = 13)	PG2 (n = 13)	p value
Dopamine receptor blockers	0 (0.0)	3 (23.1)^a^	5 (38.5)^a^	< 0.01
Platelet anti-aggregate	5 (20.8)	4 (30.8)	2 (15.4)	0.62
Antiarrhythmic	0 (0.0)	1 (7.7)	2 (15.4)	0.16
Anticholinergic	0 (0.0)	0 (0.0)	2 (15.4)	0.05
Antidepressants	1 (4.2)	4 (30.8)	4 (30.8)	0.05
Beta blocker	2 (8.3)	5 (38.5)	0 (0.0)^b^	0.01
Biguanides	4 (16.7)	3 (23.1)	2 (15.4)	0.85
Ca + channel blocker	2 (8.3)	1 (7.7)	1 (7.7)	0.99
Angiotensin II blockers	10 (41.7)	6 (46.2)	3 (23.1)	0.42
Ciprofibrate	0 (0.0)	1 (7.7)	0 (0.0)	0.23
Amantadine hydrochloride	0 (0.0)	2 (15.4)	2 (15.4)	0.13
Diuretic	6 (25.0)	2 (15.4)	2 (15.4)	0.69
Entacapone	0 (0.0)	1 (7.7)	1 (7.7)	0.38
Statins	7 (29.2)	3 (23.1)	3 (23.1)	0.88
Gliclazide	2 (8.3)	2 (15.4)	0 (0.0)	0.35
ACE inhibitor	3 (12.5)	0 (0.0)	0 (0.0)	0.17
MAO inhibitor	0 (0.0)	2 (15.4)	2 (15.4)	0.13
Levodopa	0 (0.0)	9 (69.2)^a^	9 (69.2)^a^	< 0.01
Other	16 (66.7)	7 (53.8)	11 (84.6)	0.23
Vasodilator	1 (4.2)	3 (23.1)	1 (7.7)	0.17

a Value with difference in relation to control group; ^b^value with difference in relation to PG1; CG: control group; PG1: Parkinson group below the median; PG2: Parkinson group above the median; n (percent); Ca^+^: calcium; ACE: angiotensin-converting enzyme; MAO: monoamine oxidase.

Comparisons of linear indices in the time and frequency domains between the control group (CG) and Parkinson groups (below the median - PG1; and above the median - PG2) can be observed in Tables 3 and 4, respectively. PG1 and PG2 presented statistically significant reductions in rMSSD, SDNN, pNN50, LFms^2^ and HFms^2^, compared with CG (p < 0.05). No significant differences were found between the groups regarding Mean RR, LFun, HFun and LF/HF ratio (p > 0.05).

**Table 3.** Comparison of the heart rate variability indices in the time domain between the control group (CG) and Parkinson’s groups, divided by the length of time since diagnosis (below the median - PG1; and above the median - PG2).

	CG (n = 24)	PG1 (n = 13)	PG2 (n = 13)	p value	ES	EF
Mean RR (ms)	957.81 ± 87.96 920.67-994.95	972.08 ± 142.67 885.87-1058.30	1016.09 ± 157.29 921.04-1111.14	0.24	0.061	Low
SDNN (ms)	26.20 ± 11.72 21.25-31.14	14.10 ± 5.07^a^ 11.03-17.17	14.63 ± 6.01^a^ 11.00-18.27	< 0.001	0.302	High
rMSSD (ms)	24.48 ± 10.29 20.13-28.83	14.92 ± 6.08^a^ 11.25-18.59	15.30 ± 6.49^a^ 11.39-19.22	0.001	0.258	High
pNN50	4.95 [9.20] 3.27-9.17	0.80 [1.45]^a^ 0.30-1.92	0.80 [1.06]^a^ 0.29-1.57	0.003	0.229	High

Mean ± standard deviation; lower boundary - upper boundary of 95% confidence interval; median [interquartile range]; ^a^value with difference in relation to CG (p < 0.05); ES: eta squared; EF: effect size; CG: control group; PG1: Parkinson group 1; PG2: Parkinson group 2; mean RR: RR interval mean; SDNN: standard deviation of all normal RR intervals, expressed in milliseconds; RMSSD: root mean square of differences between adjacent normal RR intervals in a time interval, expressed in ms; pNN50: percentage of adjacent RR intervals with a difference in duration > 50 ms.

**Table 4.** Comparison of the heart rate variability indices in the frequency domain between the control group (CG) and Parkinson’s groups, divided by the length of time since diagnosis (below the median - PG1; and above the median - PG2).

	CG (n = 24)	PG1 (n = 13)	PG2 (n = 13)	p value	ES	EF
LF (nu)	60.95 ± 17.51 53.56-68.35	60.25 ± 17.27 49.82-70.69	53.68 ± 16.65 43.61-63.74	0.53	0.028	Low
HF (nu)	38.95 ± 17.46 31.58-46.33	39.62 ± 17.21 29.22-50.01	46.20 ± 16.58 36.18-56.22	0.53	0.028	Low
LF (ms^2^)	309.00 [461.25] 246.63-586.87	62.00 [113.00]^a^ 35.04-179.73	95.38 ± 72.22^a^ 51.74-139.03	0.003	0.230	High
HF (ms^2^)	181.50 [231.50] 145.99-317.76	38.00 [90.50]^a^ 32.68-99.63	90.31 ± 74.61^a^ 45.22-135.39	0.004	0.219	High
LF/HF (ms^2^)	2.09 ± 1.38 1.50-2.67	2.03 ± 1.36 1.21-2.84	1.35 [1.03] 0.72-2.39	0.65	0.019	Low

Mean ± standard deviation; lower boundary - upper boundary of 95% confidence interval; median [interquartile range]; a value with difference in relation to CG (p< 0.05); CG: control group; PG1: Parkinson group 1; PG2: Parkinson group 2; ES: eta squared; EF: effect size; LF: low frequency; HF: high frequency; nu: normalized unit; ms2: milliseconds squared.

## DISCUSSION

The results obtained through the linear HRV indices suggest that the length of time since diagnosis did not influence the cardiac autonomic modulation of individuals with PD. However, individuals with PD presented reduced global variability and parasympathetic modulation, compared with individuals without the disease.

This study predominantly included men and older adults, with cardiovascular risk factors such as overweight and pre-hypertension. It is known that the incidence of PD is higher among men^29^ and individuals over 65 years of age^30^, and that overweight and obesity are common among individuals with PD^31^ and older adults without the disease^32^. Furthermore, blood pressure abnormalities can occur in the early stages of PD^33^, as observed in our patients. Thus, we consider that the participants in this study represented the reality found in the general population^29^ ^-^ ^31^ ^,^ ^33^.

Differences in the length of time since diagnosis were found between the PD groups. This was normal and expected according to the division of groups proposed in this study. Furthermore, statistical differences relating to the MMSE were found, but we do not consider that these differences were clinically important, because the individuals were classified according to their degree of schooling.

 Regarding cardiac autonomic modulation, the rMSSD, pNN50 and HFms^2^ indices that reflect parasympathetic modulation^7^ were lower in both PD groups than in the CG, with a high effect size. These results demonstrate that parasympathetic modulation is reduced among individuals with PD, thus suggesting that the presence of PD is more important than the length of time since diagnosis, with regard to affecting parasympathetic modulation. This corroborates the findings of Rocha et al.^10^, who reported that the rMSSD index was lower among individuals with PD than among those without the disease, thus indicating reduced parasympathetic modulation in these individuals. However, that study did not consider the influence of the length of time since diagnosis between individuals with PD, unlike the current study.

A reduction in parasympathetic modulation is associated with increased risks of mortality and morbidity, and with development of some risk factors^34^ and can be a sign for predicting cardiovascular and metabolic health^13^. These results emphasize the importance of pharmacological and non-pharmacological interventions, such as aerobic exercise^35^, among individuals with PD, regardless of the length of time since diagnosis, in order to promote better autonomic parasympathetic modulation response and mitigate possible damage to the organism, such as manifestation of gastrointestinal malfunction, cardiovascular dysregulation, urinary disturbance or sexual dysfunction^36^.

The global variability represented by the SDNN index is reduced in individuals with PD, regardless of the length of time since diagnosis, in comparison with individuals without the disease. Studies have shown that the reduction in the SDNN index can occur at the beginning of the disease, thus indicating involvement of the ANS physiology^37^. Ke et al.^3^ ^8^ also demonstrated that a significant reduction in global variability occurred among individuals with PD, compared with individuals without the disease. Those authors reported SDNN values of 45.50 ms for the control group and 34.50 ms for the Parkinson group, which were higher than the values found in the current study, which were 26.19 ms for the control group, 14.10 ms for the group with shorter time since diagnosis and 14.63 ms for the group with longer time since diagnosis. The duration of the HRV analysis may explain these differences, since it was 24 hours in the study by Ke et al.^38^ and 30 minutes in the current study. In addition to evaluation of the length of time since diagnosis, our study also suggests that these differences can be identified with less duration of analysis, which is clinically important.

Parasympathetic modulation and HRV reduction have been shown to present vagal sympathetic imbalance^39^ in subjects with PD. This could be caused by degeneration of the central and autonomic nervous system interaction regions, such as the hypothalamus, dorsal vagal nucleus, nucleus ambiguous, postganglionic sympathetic neurons in the pre-vertebral region and paravertebral ganglia, and in the dopaminergic nigrostriatal pathway^37^. This HRV reduction also demonstrates insufficient ANS adaptation^7^.

No differences were observed between the groups with regard to LFun, HFun and the LF/HF ratio. These results were expected since these indices are calculated from the power spectrum area and a reduction in these spectra is found in individuals with PD, when analyzed in ms^2^. As these indices are normalized with regard to the power spectrum area, no differences are observed. The reduced LFms^2^ and HFms^2^ in individuals with PD, with a high effect size, also explains the absence of significant differences in the LF/HF ratio between the groups. 

The RR interval analysis has a relationship with HR values, and no differences between the groups were observed in relation to either index. These results corroborate those of Soares et al.^39^, who also observed reduced parasympathetic and HRV indices with no significant HR reduction^39^. These results are in agreement, particularly because HRV is observed in terms of precise units of time that present greater sensitivity than HR values. 

Reduced LFms^2^ was observed in individuals with PD in comparison with the control group. Given the association with reduced parasympathetic and global modulation, this result may suggest that individuals with PD have increased sympathetic modulation, as reported by other authors^17^. Nevertheless, the data in the literature are divergent regarding the predominance of high sympathetic modulation quantified through the LF index^40^. In this regard, we take the view that further studies are needed in order to evaluate sympathetic modulation directly, in order to confirm any alterations among individuals with PD. 

To complete the information discussed above, the use of medicines should be considered to be a limitation. Nevertheless, we described all the medicines used in detail, and only a few differences were observed. Statistically significant differences were observed with regard to DBP, which could be related to the difference found in beta-blocker medication. It is also important to emphasize that due to the average age of our participants, it was common for them to use drugs to control risk factors, which reflects the reality of this population. Two other differences were found, one in relation to dopamine receptor blockers, which are medicines for psychiatric treatment, and the other to Levodopa, which is specific medication for PD treatment. To minimize this limitation, all participants with PD were evaluated during the “on” period of Levodopa. Moreover, the length of time since diagnosis was defined through analysis on medical records, which may represent a source of error, since these patients may have started to feel the symptoms before seeking a clinic to obtain the diagnosis. Despite the limitations, it is important to highlight the originality of this study. Although there was already some information in the literature about factors that might influence the cardiac autonomic modulation of other populations^11^ ^-^ ^14^, or even factors such as the stage of the disease, specifically with regard to PD^15^, this was the first study to investigate the influence of the length of time since diagnosis on the cardiac autonomic modulation of individuals with PD. This is important because this time period has a relationship with the damage caused by this degenerative disease.

In summary, our results suggest that the presence of PD, regardless of the length of time since diagnosis, can influence cardiac autonomic modulation. Furthermore, individuals with PD present reductions in global and parasympathetic modulation, compared with individuals without the disease. These issues emphasize the need for prevention and treatment among individuals with PD, along with the importance of aerobic exercise interventions^10^, which may promote increased HRV among individuals with PD, independent of the length of time since diagnosis.

In conclusion, the length of time since the diagnosis of PD was made did not influence cardiac autonomic modulation. However, PD promotes reductions in parasympathetic modulation and global variability.
